# Evaluation of BMI‐based tube voltage selection in CT colonography: A prospective comparison of low kV versus routine 120 kV protocol

**DOI:** 10.1002/acm2.13955

**Published:** 2023-03-10

**Authors:** Beibei Li, Xu Wang, Yong Fan, Shigeng Wang, Xiaoyu Tong, Jingyi Zhang, Jianying Li, Yijun Liu

**Affiliations:** ^1^ First Affiliated Hospital of Dalian Medical University Dalian China; ^2^ GE Healthcare China Dalian China

**Keywords:** colon, computed tomography

## Abstract

**Aim:**

To explore the value of individualized kVp selection based on the patient's body mass index (BMI, kg/m^2^) in CT colonography (CTC).

**Materials and Methods:**

Seventy‐eight patients underwent two CTC scans: conventional 120 kVp in supine position (Group A) with 30% Adaptive statistical iteration algorithm (ASIR–V) and BMI–based lower kV p in prone position (Group B): tube voltage was suggested by an experienced investigator according to the patient's body mass index (BMI; calculated as weight divided by height squared; kg/m (2)).70 kV for BMI < 23 kg/m^2^ (Group B1, *n* = 27), 80 kV for 23 ≤ BMI ≤ 25 kg/m^2^(Group B2, *n* = 21) and 100 kV for BMI > 25 kg/m^2^ (Group B3, *n* = 30). Group A, corresponding to the BMI value in Group B, was divided into A1, A2, and A3 subgroups for analysis. Groups B used ASIR‐V of different weights (30%–90% ASIR–V). The Hounsfield Unit (HU) and SD values of the muscles and the intestinal cavity air were measured, and the signal‐to‐noise ratio (SNR) and the contrast‐to‐noise ratio (CNR) of images were calculated. Imaging quality was evaluated by two reviewers and statistically compared.

**Results:**

The 120 kV scans were preferred more than 50% of the time. All images had excellent quality with good consistency between reviewers (Kappa > 0.75, *p* < 0.05). The radiation dose was reduced in groups B1, B2 and B3 by 63.62%, 44.63%, and 32.14%, respectively, compared with group A (*p* < 0.05). The SNR and CNR values between group A1/A2/A3 and B1/B2/B3 + 60%ASIR‐V were not statistically significant (*p* < 0.05). There was no statistically significant difference between the subjective scores of group B combined with 60%ASIR‐V and group A (*p* > 0.05).

**Conclusion:**

BMI‐based individualized kV CTC imaging significantly reduces overall radiation dose while providing an equal image quality with the conventional 120 kV.

## 1 INTRODUCTION

Colorectal cancer (CRC) is one of the most common and deadly malignant tumor types. Its incidence is rising in some countries where screening and prevention programs are not routinely undertaken.^1^, ^2^, ^3^ Despite the good diagnostic performance of colonoscopy screening, some patients, especially elderly patients, cannot tolerate the procedure due to anxiety, fear, and discomfort. Although the overall complication rate is low, the potential risk of perforation and bleeding cannot be ignored during the colonoscopy examination. The literature showed that CT colonography (CTC) has progressively evolved into a validated assessment for colorectal diseases.^4^ Its capacity of identifying CRC and large polyps in symptomatic and asymptomatic individuals is almost equivalent to a conventional colonoscopy. CTC is considered the best surgical positioning and navigation method because it is a safe and non‐invasive examination that can detect lesions both inside and outside the intestine.^5^, ^6^, ^7^ Moreover, CTC could display the whole colorectum from multiple directions and angles through two‐dimensional (2D) and three‐dimensional (3D) reconstructed images.^8^, ^9^ The main disadvantage of CTC is the potential damage of ionizing radiation,^10^, ^11^ CTC examination requires two scans in the supine and prone positions, with a more extensive scanning range from the apex of the diaphragm to the pubic joint. Therefore, keeping the dose as low as possible without significantly sacrificing image quality is strongly advised. There is a naturally high contrast between the air in the intestine and the soft tissues of the intestinal wall, allowing for low‐dose CTC scanning. The effect of low tube voltage on CTC image quality has been evaluated.^12^ In addition, Chang et al.’s research showed that 100 kV combined with FBP algorithm CTC imaging could reduce volume CT dose index (CTDIvol) by 20% and reduce dose‐length product (DLP) by 16%.^13^ Reduced tube voltage can increase image noise, leading to poor image quality that will affect the detection of small lesions. Therefore, this study adds the post‐weight ASIR‐V algorithm to reduce the noise while reducing the radiation dose, thus addressing the image quality problem caused by the tube voltage reduction. Therefore, our study aimed to optimize the scanning voltages in CTC for all patients, and to evaluate the use of individualized kV (70 kV, 80 kV, 100 kV) combined with the optimal weight of ASIR‐V protocol to reduce the patient's radiation dose, contributing to the establishment of BMI‐based individualized CTC imaging protocols.

## 2 MATERIALS AND METHODS

### 2.1 Participants

Patients who underwent CT Colonoscopy (CTC) examination from March 2021 to May 2022 were enrolled prospectively. The study protocol was conducted in accordance with the Declaration of Helsinki and was approved by the Institutional Review Board. We also recommended the guideline to the China Clinical Trials Registration Platform, the WHO Clinical Trials Registration Institution, and the National Health Commission of the People's Republic of China. The exclusion criteria were set as follows: incomplete colonoscopy (*n* = 48), inadequate bowel preparation (*n* = 20), and inability to remove metal artifacts from the body trunk (*n* = 2). Each patient's demographic data, including age, gender, height, weight, and Body Mass Index (BMI), were collected at admission.

### 2.2 Preparation before scanning

All participants performed the standard bowel preparation, which comprised of three‐meal low‐residue diets before the day of CTC and the administration of laxatives which included magnesium sulfate, senna leaf, and ricinoleic acid before the examination. The smooth muscle of the abdomen was under relaxation with spasmolytics to compensate for the movements of the contents in the gastrointestinal tract. According to the ESGE guidelines, adequate bowel preparation should result in the absence of visible residual stool or fluid in the colon. Repeated colonoscopy on the following day (after further colon cleaning) was recommended in patients with insufficient bowel preparation. After completing all preparations, when patients were in the left lateral position with their hips and knees flexed to 90 degrees, a radiologist began insufflation of gas into the low rectum via a thin rectal catheter until the patient experienced mild discomfort. Considering comfort of patients and gas of completeness, the entire procedure was monitored via scout scan to ensure adequate colonic distension. The total volume of gas was approximately 1−2 L.^14^ If the image quality was nonoptimal, additional gas was insufflated.

### 2.3 CT examination and data reconstruction

All patients were scanned twice in the prone and supine positions using a 256‐row CT scanner (Revolution CT, GE Healthcare, USA). In the supine position, group A received a standard dose with 120 kV and 30% ASIR‐V reconstruction. In the prone position, tube voltage was suggested by an experienced investigator according to the patient's body mass index (BMI; calculated as weight divided by height squared; kg/m (2)). In our study, participants were categorized in three groups: A1 and B1(BMI < 23), A2 and B2 (23 ≤ BMI ≤ 25), A3 and B3 (BMI > 25). In each subgroup, Groups A and B shared the same patient.70 kV was chosen for patients with a BMI under 23 kg/m^2^, 80 kV was chosen for those with a BMI between 23 and 25 kg/m^2^, and 100 kV was used for those with a BMI greater than 25 kg/m^2^, with reconstruction ranging from 30% to 90% ASIR‐V. (10 percent interval). The remaining parameters remained constant (Table 1). A scan was done from the diaphragm to the pubic symphysis. CT volume dose index (CTDI_vol_) and dose‐length product (DLP) were recorded for each group. Effective radiation dose (in milliseiverts) was estimated by using the dose‐length product multiplied by a conversion coefficient for the abdomen (*k* = 0.015 mSv mGy ^−1^ cm ^1^). 0.625 mm slice interval reconstructed images were sent to the AW 4.6 workstation. Radiologists reconstructed images using the CT Colon VCAR software, which included 2D axial images, multi‐planar reconstruction (MPR) images, three‐dimensional (3D) endoluminal images, and the colon's raysum.^15^

**TABLE 1:** CT parameters.

	Individualized scan protocol		Routine protocol
Scanner model	Revolution CT		
Position	Prone		Supine
Tube potential(kV)	BMI (kg/cm^2^) < 23 (B1, *n* = 27)	70	120 (A1)
	23 ≤ BMI (kg/cm^2^) ≤ 25 (B2, *n* = 21)	80	120 (A2)
	BMI (kg/cm^2^) > 25 (B3, *n* = 30)	100	120 (A3)
Tube current modulation	Smart‐mA		Smart‐mA
Pitch	0.992		0.992
Tube rotation time	0.5s		0.5s
Collimation	0.5 × 80 mm		0.5 × 80 mm
Slice thickness	5 mm		5 mm
noise index (NI)	13HU		13HU
Reconstruction kernel	Standard		Standard
Reconstruction algorithm	ASIR‐V 30%−90%		ASIR‐V 30%
Reconstruction thickness	0.625 mm		0.625 mm

### 2.4 Quantitative image analysis

On the axial image, the circular regions of interest (ROIs) were placed manually by the observer at three levels of uniform density on both the psoas major muscle and the air of the intestinal cavity in the same slicer, and HU values and SD values were measured and the averaged value of the three image levels were calculated keeping the ROI size around 100 mm^2^. The ROI was placed in the same position as the prone and supine datasets by using the “copy‐paste” tool. The standard deviation of the air in the intestinal cavity was used as the background noise value. Finally, the signal‐to‐noise ratio (SNR) and contrast‐to‐noise ratio (CNR) were calculated.

### 2.5 Qualitative image analysis

Two radiologists with over 10 years of diagnostic experience scored the CTC axial images and reconstructed images on a five‐point scale using a double‐blind method. The following criteria were demonstrated^16^:

- 5 points: virtually no artifact
- 4 points: low image noise and the presence of a few artifacts
- 3 points: images with a moderate amount of noise and minor artifacts
- 2 points: a high level of image noise and a moderate level of artifacts
- 1 point: the highest level of noise plus the most obvious artifacts

Different criteria were applied to 3D reconstruction images.

- 5 points: a very smooth bowel wall, visible mucosal folds, sharp edges, and a clear lesion morphology.
- 4 points: the bowel wall is less smooth, the mucosal folds are less clear, the sharp edges are lost, and the lesion morphology is clear.
- 3 points: a slightly rough bowel wall and imprecise mucosal folds with blurred edges. The morphology and structure of the lesion are visible, which does not affect diagnosis.
- 2 points: the diagnosis is hampered by the rough intestinal wall, blurry mucosal folds and edges, and unclear lesion morphology.
- 1 point: an extremely rough intestinal wall with imprecise mucosal folds and indistinguishable edges, as well as an indistinct lesion morphological structure.

In both evaluations, images with scores less than 3 were considered non‐diagnostic.

### 2.6 Subjective analysis

Subsequently, paired images were presented to the radiologists using either 120 kV combined with 30% ACIR‐V or with low kV and varying weights of ASIR‐V. Each radiologist was asked to designate for each pair, viewed side by side:

- the consistency of the lesions' location in CTC with the colonoscopy results, which were used as the standard;
- which weight of ASIR‐V (low‐kV protocol) provided the identical image quality with the 120 kV;
- the detection of lesions (polyps <5 mm, polyps ≥5 mm, adenoma, LST and, malignant tumor), if detected, on which protocol (120 kV, low‐kV, or both) these lesions were better identified/analyzed. Only lesions detected and analyzed by both reviewers were ultimately considered.

### 2.7 Statistical analysis

The data were processed using SPSS 24.0 statistical software. X ± S was used to represent the one‐way analysis of variance (ANOVA) for variables with a normal distribution. The Kruskal‐Wallis H test was used to analyze variables with non‐normal distribution. ANOVA with repeated measures was used to compare the SNR and CNR of ASIR‐V images with varying weights. The Kappa test was utilized to estimate the consistency of two observers' subjective quality scores for the images. The Kruskal‐Wallis H test was used to compare the radiation dose and the subjective quality scores. *p* < 0.05 was considered statistically significant.

## 3 RESULTS

### 3.1 Population

**FIGURE 1:**
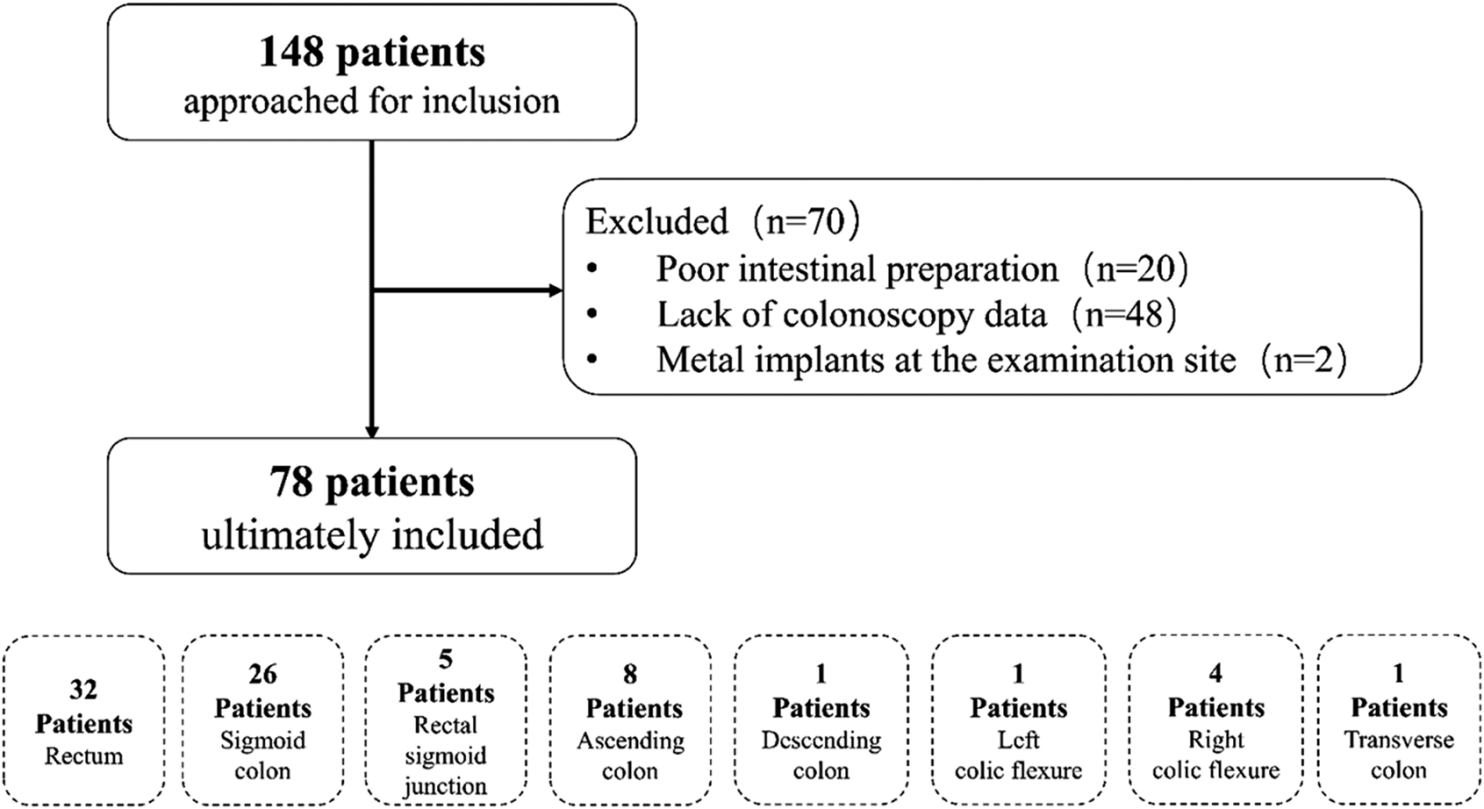
Flowchart of the study.

148 patients were approached, and 70 were excluded from the study. Seventy‐eight patients were ultimately included (Figure 1), including 42 men and 36 women, with a mean age of 62.65 ± 1.12 (SD) years (range: 30−82 years). The mean height was 167.44 ± 0.88 (SD) cm (range: 151−185 cm). The mean weight was 67.68 ± 1.43 (SD) kg (range: 45−104 kg). The mean BMI was 23.88 ± 0.41 (SD) kg/m^2^ (range: 17.15−34.35 kg/m^2^).

### 3.2 Consistency of the lesions' location CTC/colonoscopy

All 78 patients had successful CTC examination and no repeated scans were needed. All the 78 patients underwent colonoscopy before or after the CTC examination. Colonoscopy showed that lesions in eight patients were in the ascending colon (10.26%), four in the right colic flexure of the colon (5.13%), one in the transverse colon (1.28%), one in the left colic flexure (1.28%), one in the descending colon (1.28%), 26 cases in the sigmoid colon (33.33%), five cases in the rectal sigmoid junction (6.41%), and 32 cases in the rectum (41.03%). Three patients’ results of the 70 kV CTC scan protocol in the prone position with ultralow rectal cancer did not match with those of colonoscopy, and the other 24 patients were all consistent with colonoscopy. The results of CTC with 80 kV also showed that three patients with the rectal sigmoid junction did not agree with the results of colonoscopy. In 100 kV, the results showed that one patient with the ascending colon and two patients with rectum did not agree with the results of colonoscopy. Compared with colonoscopy, individualized low‐kV CTC scan protocol had higher intrinsic consistency in confirming lesion’ location.

### 3.3 Radiation dose

The average CTDI_vol_, DLP, and ED of group B as a whole were decreased by 43.90%, 46.89%, and 46.89% in comparison with group A. In the subgroup analysis of the degree of radiation dose reduction between group A and group B, compared with the group A1–A3, CTDI_vol_ in group B1–B3 decreased by 62.11%, 42.64%, and 27.89% respectively. DLP decreased by 63.62%, 44.63%, and 32.14% and ED decreased by 63.62%, 44.63%, and 32.14% in group B1–B3, respectively (all *p* < 0.05, Table 2).

**TABLE 2:** Radiation dose in individual low kVp group and routine 120 kVp group.

Group	*N*	BMI (kg/cm^2^)	kVp	CTDI_vol (_mGy)	DLP (mGy × cm)	ED (mSv)	*p*
A1	27	<23	70	1.58 ± 0.03	75.64 ± 1.77	1.15 ± 0.03	< 0.05
B1			120	4.17 ± 0.08	207.93 ± 5.23	3.21 ± 0.09	
A2	21	23–25	80	2.69 ± 0.06	130.73 ± 3.29	2.14 ± 0.08	<0.05
B2			120	4.69 ± 0.11	236.1 ± 6.27	3.92 ± 0.13	
A3	30	>25	100	4.86 ± 0.16	241.92 ± 8.54	3.57 ± 0.20	<0.05
B3			120	6.74 ± 0.16	356.6 ± 10.48	5.23 ± 0.49	

### 3.4 Quantitative image analysis

**FIGURE 2:**
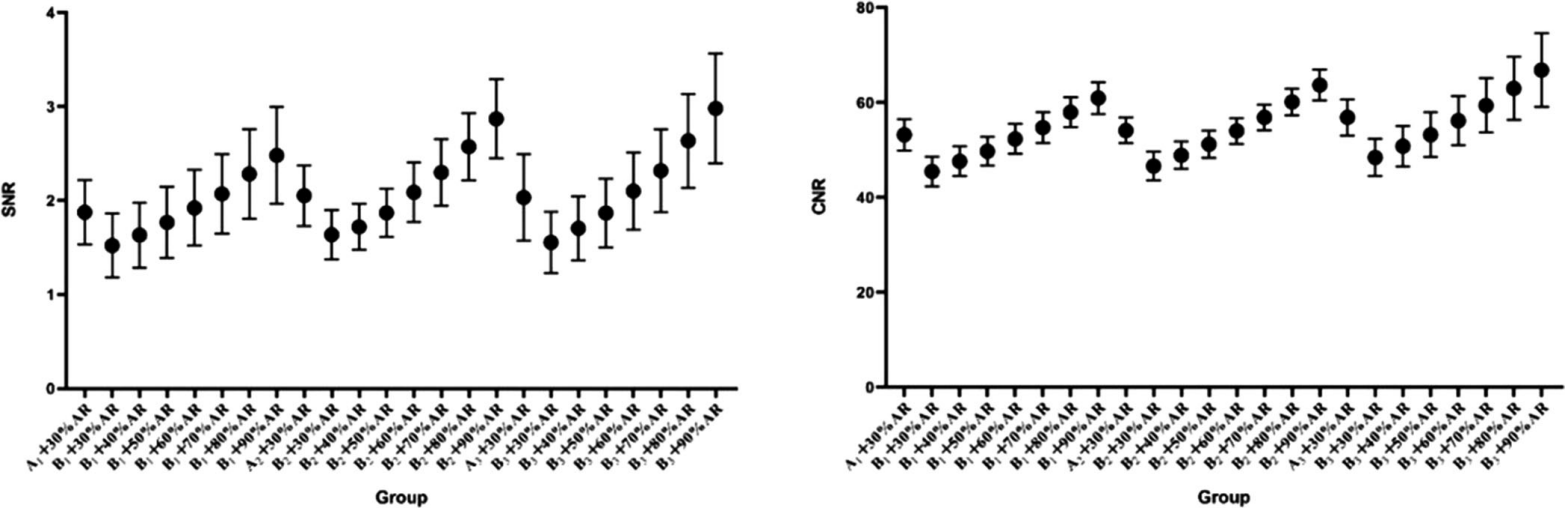
Comparison of SNR and CNR of the images.

The SNR and CNR increased with the increase of ASIR‐V weights (*p* < 0.05) in groups. There was no significant difference between group B1 + 60% ASIR‐V SNR (1.92 ± 0.40 HU) and CNR (52.32 ± 3.14 HU) and group A1 (1.88 ± 0.34 HU) and CNR (53.21 ± 3.29 HU). There was no significant difference between group B2 + 60% ASIR‐V SNR (2.05 ± 0.32 HU) and CNR (54.04 ± 2.71 HU) and group A2 (2.09 ± 0.32 HU) and CNR (54.12 ± 2.69 HU). There was no significant difference between B3 + 60% ASIR‐V SNR (2.10 ± 0.41 HU) and CNR (56.19 ± 5.22 HU) and group A3 (2.03 ± 0.46 HU) and CNR (56.89 ± 3.80 HU, Figure 2)

### 3.5 Qualitative image analysis

The subjective scores of the two observers on the image quality of each group were consistent (*Kappa* value = 0.684–0.907), and there was no statistically significant difference between the subjective scores of group B combined with 60% ASIR‐V and group A (*p* > 0.05, Table 3). The SNR and CNR of group A1, A2, and A3 were higher than those of group B1, B2, and B3 below 60%  ASIR‐V, respectively, and the difference was statistically significant (all *p* < 0.001), while the SNR between group A1/A2/A3 and B1/B2/B3 + 60%ASIR‐V was not statistically significant (*p* > 0.05). There was no significant difference in the diagnostic confidence of 2D‐images and 3D‐images between group A1/A2/A3 and B1/B2/B3 + 60% ASIR‐V (all *p* ≥ 0.05). The 120 kV acquisition was preferred over the 80 kV for 100%  of patients for both readers. Eighty‐seven lesions in group A1 and B1 were detected and analyzed by both readers. Depending on the reader, Lesions were better seen on the 120 kV acquisition in 59.8% (52/87 lesions) and 56.3% (49/87 lesions) of examinations and were equally seen on both acquisitions in 40.2% (35/87 lesions) to 43.7% (38/87 lesions) of patients. For no lesions the 70 kV protocol provide better depiction than the 120 kV protocol. Fifty‐one lesions in group A2 and B2 were detected and analyzed by both readers. Depending on the reader, lesions were better seen on the 120 kV acquisition in 56.9% (29/51 lesions) and 58.8% (30/51 lesions) of examinations and were equally seen on both acquisitions in 43.1% (22/51 lesions) to 41.2% (21/51 lesions) of patients. For no lesions the 80 kV protocol provided a better depiction than the 120 kV protocol. Sixty‐three lesions in group A3 and B3 were detected and analyzed by both readers. Depending on the reader, lesions were better seen on the 120 kV acquisition in 49.2% (31/63 lesions) and 50.8% (32/63 lesions) of examinations and were equally seen on both acquisitions in 50.8% (32/63 lesions) to 49.2% (31/63 lesions) of patients. For no lesions the 100 kV protocol provided a better depiction than the 120 kV protocol.

**TABLE 3:** Comparison of image quality marks (Scores:x ± s).

Group	*n*	2D images analysis				3D images analysis			
		Reader 1	Reader 2	Kappa	*p*	Reader 1	Reader 2	Kappa	*p*
A1	27	3.85 ± 0.53	3.81 ± 0.48	0.917	0.262	4.11 ± 0.50	4.18 ± 0.55	0.847	0.208
B1 + 60% ASIR‐V		4.00 ± 0.48	4.00 ± 0.48	0.812		4.26 ± 0.45	4.29 ± 0.47	0.907	
A2	21	4.14 ± 0.48	4.05 ± 0.38	0.720	0.576	4.38 ± 0.59	4.38 ± 0.59	0.836	0.186
B2 + 60% ASIR‐V		4.19 ± 0.40	4.09 ± 0.30	0.618		4.52 ± 0.51	4.57 ± 0.51	0.712	
A3	30	4.76 ± 0.43	4.70 ± 0.47	0.831	0.735	4.73 ± 0.45	4.67 ± 0.48	0.684	0.463
B3 + 60% ASIR‐V		4.73 ± 0.45	4.77 ± 0.43	0.734		4.67 ± 0.48	4.90 ± 0.50	0.714	

## 4 DISCUSSION

Colonoscopy is a preferred screening method for colorectal cancer and can be intervened during screening. However, colonoscopy is an invasive procedure requiring high tolerance, sedation, or analgesia.^17^ On the contrary, CTC is a safe, noninvasive imaging technique that provides multidimensional images of the colorectal region in the preoperative evaluation of colorectal diseases.^18^ Although several papers have been published, dose reduction and optimization in CT imaging still require consistent efforts. Over the past few years, various technological innovations have been introduced, such as bow‐tie filter,^19^ low‐tube voltage scanning,^20^ and iterative image reconstruction,^21^ which steadily made efforts to reduce radiation dose. Questions such as how these techniques affect colorectal structural visibility and what is the best option for achieving a low radiation dose in CTC, remain unclear.

According to the principle of as low as reasonably achievable (ALARA), CT acquisition parameters should be optimized based on the patient's character to reduce radiation dose while meeting diagnostic requirements. Most studies have focused on middle‐sized individuals rather than overweight or obese people when developing dose‐reduction techniques.^22^ To homogenize our findings in a population involving obese patients, we conducted three degrees of kV according to patients' body sizes. Unlike previous studies, this study uses BMI as the basis of individual acquisition parameters. In other words, our study selects the appropriate tube voltage according to the patient's BMI, followed by the optimal weight iterative reconstruction. Most previous studies reduced the radiation dose by decreasing mAs as the radiation dose has a linear relationship with mAs.

In contrast, the radiation dose has an exponential relationship with kV, so decreasing kV is more efficient in reducing the radiation dose. Low kV scanning can significantly increase image noise, while post‐ASIR‐V can reduce image noise even further. The square of tube voltage is approximately inversely proportional to the radiation dose, and lower tube voltage could reduce the radiation dose more effectively, which has been demonstrated that it could been successfully used in imaging techniques such as CTA.^23^, ^24^, ^25^ Some studies showed that kVp may be selected based on body size (body mass index, body width on scout topogram, or patient weight),^26^ for example, 100 kVp can be used for patients weighing less than 150 lbs. In our study, the mean weight was 67.68 ± 1.43 (SD) kg (150lbs = 68.0388555 kg). Our study selected suitable tube voltage and the optimal weight in iterative reconstruction based on BMI to carry out individualized low‐dose CTC. Group A used conventional scanning with tube voltage 120 kVps, while group B according to the difference in BMI of patients, selected low tube voltage (70 kVp, 80 kVp, 100 kVp) for low‐dose scanning. Post‐ASIR‐V has 11 weights ranging from 0% to 100%, so ASIR‐V was included in this study. Adaptive Statistical Iterative Reconstruction (ASIR‐V) is a rapid reconstruction technology at either the front‐end or back‐end. The former ASIR‐V reduces radiation dose through an intelligently regulated tube current, and the post‐ASIR‐V had significant effects on noise reduction, contrast improvement, and artifacts elimination. There was no standard for the optimal weight of ASIR‐V in optimized dose CTC images.

**FIGURE 3:**
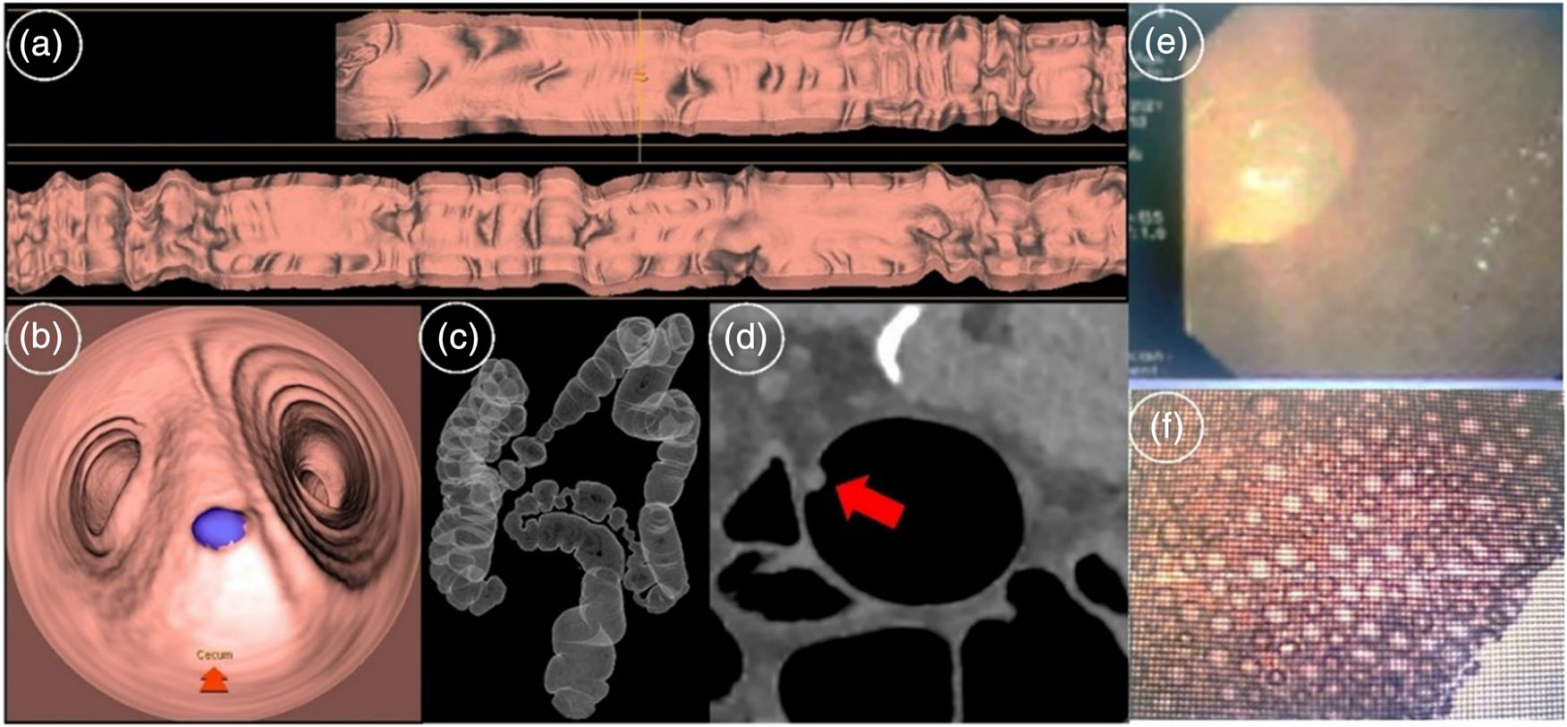
Female, aged 71, height 1.65 m, weight 68 kg, BMI: 24.9 kg/m^2^, rectal polyps with a diameter of 3 mm; (a–d) CTC display; parameters: prone, 80 kV, 60% ASIR‐V; (e) colonoscopy display; (f) pathological examination display.

Furthermore, this study showed that individualized kV acquisition parameters based on the patient's BMI, combined with the optimal ASIR‐V weight, had no significant impact on the 2D and 3D image quality and the diagnosis of colorectal diseases (Figure 3).^27^ One advantage of ASIR‐V reconstruction was low image noise, directly affecting image quality. The image with individualized low kV and high weight ASIR‐V had the lowest noise, while the 2D axial image had the lowest score. In addition, the reduction of image noise was not necessarily accompanied by the improved 3D reconstructed image quality. In our study, subgroup analysis showed that low kV combined with the best weight ASIR‐V protocol had an equal image quality with the conventional 120 kV combined with fixed weight ASIR‐V protocol.

In a phantom study, Cohnen et al. assessed image quality and sensitivity at ultra‐low radiation doses by using ultra‐low tube currents to observe detectable colorectal polyps at 1 mSv doses.^28^ The results demonstrated that the image noise decreased as ASIR‐V weighting was increased; as a result, the SNR and the CNR were improved, and the CT value remained stable. Compared with the routine 120 kV with 30% ASIR‐V images, low kV (70/80/100 kV) images showed no significant difference in the lesion morphology and could clearly show the intestinal tissue structure, which met the screening requirement of colon diseases. The DLP of the individualized CTC group decreased significantly (64.5%, 48.3%, and 33.4%, respectively), which fully proved the feasibility of CTC scanning. The lowest DLP of group B was 75.85 ± 9.75 mGy × cm, and the ED was 1.14 ± 0.15 mSv. Some studies showed that the iterative reconstruction algorithm could significantly reduce image noise and artifacts to improve detection accuracy. The application of ASIR‐V in CTC imaging was studied. It has been found that ASIR‐V could enhance imaging quality and reduce radiation dose by 50%. The researchers found that it was possible to obtain accurate quantitative CTC images that were acceptable for image noise at 1 mSv dose levels.^29^ In our study, increasing the iterative percentage of image reconstruction can significantly improve SNR and CNR. However, using a higher percentage of iterative reconstruction in the subgroup analysis of intestinal air did not significantly improve visual evaluation.

It was demonstrated that 60% ASIR‐V was the optimal ASIR‐V weight of the low kV CTC protocol. The results showed that 60% of the ASIR‐V images could compensate for the noise caused by the reduction of kV, and the image quality is comparable to that of 120 kV. The subjective score of the axial images increased steadily in the range of 30%–60% ASIR‐V but decreased after 60% ASIR‐V. The subjective scores of 3D‐reconstructed images of different ASIR‐Vs are not significantly different, mainly because the mucosal and air interfaces in the 3D‐reconstructed images are not sensitive to noise. The patient's prior intestinal preparation was the only factor that affected the image quality.^30^

Therefore, we will explore individualized scanning solutions for patients with different body indexes, and find the optimal noise index and pre‐ASIR‐V weights for low kV scanning in the future.

## 5 CONCLUSIONS

In conclusion, the application of individualized tube voltage based on the patient's BMI combined with 60% ASIR‐V technology for CTC imaging can retain good image quality, and at the same time, significantly reduce the radiation dose compared with the routine scan protocol of using a fixed 120 kV tube voltage.

## AUTHOR CONTRIBUTIONS

Conceptualization: Beibei Li and Yijun Liu; methodology: Beibei Li; formal analysis: Xu Wang; investigation: Shigeng Wang and Yong Fan; data curation: Beibei Li; writing—original draft preparation: Yijun Liu. writing—review and editing: Beibei Li; visualization: Jingyi Zhang and Jianying Li; supervision. All authors have read and agreed to the published version of the manuscript.

## CONFLICT OF INTEREST STATEMENT

The authors declare no conflict of interest.

## INSTITUTIONAL REVIEW BOARD STATEMENT

The study was conducted in accordance with the Declaration of Helsinki, and approved by the Institutional Review Board (or Ethics Committee) of First Affiliated Hospital of Dalian Medical University (PJ‐KS‐KY‐2019‐49).

## INFORMED CONSENT STATEMENT

Written informed consent has been obtained from the patient(s) to publish this paper.

## References

[1] Brody H . Colorectal cancer. Nature. 2015;521(7551):S1. 25970450 10.1038/521S1a

[2] Neri E , Faggioni L , Cini L , Bartolozzi C . Colonic polyps: inheritance, susceptibility, risk evaluation, and diagnostic management. Cancer Manag Res. 2010;3:17‐24. 21407996 10.2147/CMR.S15705 PMC3048090

[3] McFarland EG , Fletcher JG , Pickhardt P , et al. ACR Colon Cancer Committee white paper: status of CT colonography 2009. J Am Coll Radiol. 2009;6(11):756‐772. 19878883 10.1016/j.jacr.2009.09.007

[4] Yee J , Weinstein S , Morgan T , Alore P , Aslam R . Advances in CT colonography for colorectal cancer screening and diagnosis. J Cancer. 2013;4(3):200‐209. 23459511 10.7150/jca.5858 PMC3584833

[5] Graser A , Stieber P , Nagel D , et al. Comparison of CT colonography, colonoscopy, sigmoidoscopy and faecal occult blood tests for the detection of advanced adenoma in an average risk population. Gut. 2009;58(2):241‐248. 18852257 10.1136/gut.2008.156448

[6] Keeling AN , Slattery MM , Leong S , et al. Limited‐preparation CT colonography in frail elderly patients: a feasibility study. AJR Am J Roentgenol. 2010;194(5):1279‐1287. 20410415 10.2214/AJR.09.2896

[7] Yee J , Sadda S , Aslam R , Yeh B . Extracolonic findings at CT colonography. Gastrointest Endosc Clin N Am. 2010;20(2):305‐322. 20451819 10.1016/j.giec.2010.02.013

[8] Park SH , Yee J , Kim SH , Kim YH . Fundamental elements for successful performance of CT colonography (virtual colonoscopy). Korean J Radiol. 2007;8(4):264‐275. 17673837 10.3348/kjr.2007.8.4.264 PMC2627155

[9] de Haan MC , Nio CY , Thomeer M , et al. Comparing the diagnostic yields of technologists and radiologists in an invitational colorectal cancer screening program performed with CT colonography. Radiology. 2012;264(3):771‐778. 22771881 10.1148/radiol.12112486

[10] Cohnen M , Vogt C , Aurich V , Beck A , Haussinger D , Modder U . Multi‐slice CT‐colonography in low‐dose technique–Preliminary results. Rofo. 2002;174(7):835‐838. 12101472 10.1055/s-2002-32691

[11] Perisinakis K , Seimenis I , Tzedakis A , Papadakis AE , Kourinou KM , Damilakis J . Screening computed tomography colonography with 256‐slice scanning: should patient radiation burden and associated cancer risk constitute a major concern? Invest Radiol. 2012;47(8):451‐456. 22766908 10.1097/RLI.0b013e318250a58c

[12] Liu J , Pan W , Xue H , et al. Application of low tube voltage 70 kV and advanced modeled iterative reconstruction in the third‐generation dual‐source CT to CT colonography. Zhongguo Yi Xue Ke Xue Yuan Xue Bao. 2017;39(1):95‐100. 28270290 10.3881/j.issn.1000-503X.2017.01.016

[13] Chang KJ , Caovan DB , Grand DJ , Huda W , Mayo‐Smith WW . Reducing radiation dose at CT colonography: decreasing tube voltage to 100 kVp. Radiology. 2013;266(3):791‐800. 23264348 10.1148/radiol.12120134

[14] Iafrate F , Iannitti M , Ciolina M , Baldassari P , Pichi A , Laghi A . Bowel cleansing before CT colonography: comparison between two minimal‐preparation regimens. Eur Radiol. 2015;25(1):203‐210. 25149295 10.1007/s00330-014-3345-0

[15] Andersen K , Blondin D , Beck A , et al. [Assessment of two different software solutions for the evaluation of CT colonography]. Rofo. 2005;177(9):1227‐1234. 16123868 10.1055/s-2005-858364

[16] Nagata K , Fujiwara M , Kanazawa H , et al. Evaluation of dose reduction and image quality in CT colonography: comparison of low‐dose CT with iterative reconstruction and routine‐dose CT with filtered back projection. Eur Radiol. 2015;25(1):221‐229. 25097128 10.1007/s00330-014-3350-3

[17] Lin JS , Perdue LA , Henrikson NB , Bean SI , Blasi PR . Screening for colorectal cancer: updated evidence report and systematic review for the US preventive services task force. JAMA. 2021;325(19):1978‐1997. 34003220 10.1001/jama.2021.4417 PMC13509038

[18] Obaro AE , Burling DN , Plumb AA . Colon cancer screening with CT colonography: logistics, cost‐effectiveness, efficiency and progress. Br J Radiol. 2018;91(1090):20180307. 29927637 10.1259/bjr.20180307 PMC6350489

[19] Manduca A , Yu L , Trzasko JD , et al. Projection space denoising with bilateral filtering and CT noise modeling for dose reduction in CT. Med Phys. 2009;36(11):4911‐4919. 19994500 10.1118/1.3232004 PMC4108640

[20] Yamamura S , Oda S , Imuta M , et al. Reducing the radiation dose for ct colonography: effect of low tube voltage and iterative reconstruction. Acad Radiol. 2016;23(2):155‐162. 25872861 10.1016/j.acra.2015.03.009

[21] Taguchi N , Oda S , Imuta M , et al. Model‐based iterative reconstruction in low‐radiation‐dose computed tomography colonography: preoperative assessment in patients with colorectal cancer. Acad Radiol. 2018;25(4):415‐422. 29191684 10.1016/j.acra.2017.10.008

[22] Anupindi S , Perumpillichira J , Jaramillo D , Zalis ME , Israel EJ . Low‐dose CT colonography in children: initial experience, technical feasibility, and utility. Pediatr Radiol. 2005;35(5):518‐524. 15789249 10.1007/s00247-004-1394-2

[23] Ren Z , Zhang X , Hu Z , et al. Application of adaptive statistical iterative reconstruction‐V with combination of 80 kV for reducing radiation dose and improving image quality in renal computed tomography angiography for slim patients[J]. Acad Radiol. 2019;26(11):e324‐e332. 30655053 10.1016/j.acra.2018.12.021

[24] Jia X , Li J , Zhu S . Individualized protocol for radiation and contrast medium dose reduction in one‐stop assessment for kidney transplantation patients[J]. European journal of radiology. 2021;140:109757. 33989967 10.1016/j.ejrad.2021.109757

[25] Liu Y , Liu A , Liu L , et al. Feasibility of spectral imaging with low‐concentration contrast medium in abdominal CT angiography of obese patients[J]. Int J Clin Pract. 2016;70(9B):B37‐B43. 27577512 10.1111/ijcp.12856

[26] Chang KJ , Yee J . Low‐dose computed tomography colonography technique[J]. Radiol Clin North Am. 2018;56(5):709‐717. 30119769 10.1016/j.rcl.2018.04.008

[27] Yasuda T , Honda T , Utano K , et al. Diagnostic accuracy of ultra‐low‐dose CT colonography for the detection of colorectal polyps: a feasibility study. Jpn J Radiol. 2022. 10.1007/s11604-022-01266-1 35344130

[28] Cohnen M , Vogt C , Beck A , et al. Feasibility of MDCT colonography in ultra‐low‐dose technique in the detection of colorectal lesions: comparison with high‐resolution video colonoscopy. AJR Am J Roentgenol. 2004;183(5):1355‐1359. 15505303 10.2214/ajr.183.5.1831355

[29] Lambert L , Danes J , Jahoda J , Masek M , Lisy J , Ourednicek P . Submilisievert ultralow‐dose CT colonography using iterative reconstruction technique: a feasibility study. Acta Radiol. 2015;56(5):517‐525. 24855290 10.1177/0284185114533683

[30] Pollentine A , Mortimer A , McCoubrie P , Archer L . Evaluation of two minimal‐preparation regimes for CT colonography: optimising image quality and patient acceptability. Br J Radiol. 2012;85(1016):1085‐1092. 22422379 10.1259/bjr/22421731 PMC3587090

